# Analysis of gene expression and use of connectivity mapping to identify drugs for treatment of human glomerulopathies

**DOI:** 10.3389/fmed.2023.1122328

**Published:** 2023-03-13

**Authors:** Chen-Fang Chung, Joan Papillon, José R. Navarro-Betancourt, Julie Guillemette, Ameya Bhope, Amin Emad, Andrey V. Cybulsky

**Affiliations:** ^1^Department of Medicine, McGill University Health Centre Research Institute, Montreal, QC, Canada; ^2^Department of Electrical and Computer Engineering, McGill University, Montreal, QC, Canada

**Keywords:** autophagy, endoplasmic reticulum, glomerulonephritis, podocyte, unfolded protein response

## Abstract

**Background:**

Human glomerulonephritis (GN)—membranous nephropathy (MN), focal segmental glomerulosclerosis (FSGS) and IgA nephropathy (IgAN), as well as diabetic nephropathy (DN) are leading causes of chronic kidney disease. In these glomerulopathies, distinct stimuli disrupt metabolic pathways in glomerular cells. Other pathways, including the endoplasmic reticulum (ER) unfolded protein response (UPR) and autophagy, are activated in parallel to attenuate cell injury or promote repair.

**Methods:**

We used publicly available datasets to examine gene transcriptional pathways in glomeruli of human GN and DN and to identify drugs.

**Results:**

We demonstrate that there are many common genes upregulated in MN, FSGS, IgAN, and DN. Furthermore, these glomerulopathies were associated with increased expression of ER/UPR and autophagy genes, a significant number of which were shared. Several candidate drugs for treatment of glomerulopathies were identified by relating gene expression signatures of distinct drugs in cell culture with the ER/UPR and autophagy genes upregulated in the glomerulopathies (“connectivity mapping”). Using a glomerular cell culture assay that correlates with glomerular damage *in vivo*, we showed that one candidate drug – neratinib (an epidermal growth factor receptor inhibitor) is cytoprotective.

**Conclusion:**

The UPR and autophagy are activated in multiple types of glomerular injury. Connectivity mapping identified candidate drugs that shared common signatures with ER/UPR and autophagy genes upregulated in glomerulopathies, and one of these drugs attenuated injury of glomerular cells. The present study opens the possibility for modulating the UPR or autophagy pharmacologically as therapy for GN.

## Introduction

Human glomerular diseases, including primary glomerulonephritis (GN)—membranous nephropathy (MN), focal segmental glomerulosclerosis (FSGS) and IgA nephropathy (IgAN), as well as diabetic nephropathy (DN) are leading causes of chronic kidney disease, and have a major impact on health (1). Current therapies of GN and DN are only partially effective, significantly toxic and lack specificity. Thus, mechanism-based therapies are desirable.

Glomerular visceral epithelial cells (GECs, podocytes), mesangial and endothelial cells may all be involved in the pathogenesis of GN and DN. Among these cells, podocytes are vital in maintaining glomerular capillary wall permselectivity (2, 3) and podocyte injury is believed to be key to the pathogenesis of GN and DN. Injury may be initiated by autoantibodies to glomerular components or circulating immune complexes that deposit in glomeruli and lead to the activation of complement (MN and IgAN) (4). Alternatively, a circulating factor toxic to podocytes induces injury (FSGS) (5). In DN, hyperglycemia and oxidative stress lead to podocyte and mesangial injury (6). These distinct stimuli may activate or disturb various metabolic pathways in glomerular cells; e.g. in podocytes this results in disruption of the cytoskeleton, membrane composition and structure, adhesion to the glomerular basement membrane, or the function of organelles (due to ATP depletion). Conversely, other pathways may be activated in parallel in the glomerulus to attenuate cell injury or promote repair. These pathways may include protein kinases, cytokines, endoplasmic reticulum (ER) stress/unfolded protein response (UPR), ubiquitin-proteasome system and autophagy (7).

In the ER, secreted and membrane proteins are covalently modified (e.g., glycosylated) and attain a correctly folded conformation by the action of folding enzymes and chaperones, prior to transport to the secretory pathway (7). Intact ER function is important for protein homeostasis (“proteostasis”) in podocytes, including production of components of the slit diaphragm, focal adhesion complexes (FACs) and glomerular basement membrane (2, 3). Protein misfolding causes ER stress and activates a signaling network called the UPR (8, 9). The UPR is regulated by three transducers in the ER membrane: inositol requiring enzyme-1α (IRE1α), activating transcription factor 6 (ATF6) and protein kinase R-like ER kinase. Actions of the UPR include upregulation of ER chaperones (to enhance protein folding capacity), attenuation of mRNA translation (to reduce the protein load to a damaged ER), and degradation of misfolded proteins, e.g., *via* linkage to autophagy. There is experimental evidence for upregulation of ER chaperones and dilatation of the podocyte ER in human GN and DN, supporting a role for ER stress in disease pathogenesis (reviewed in (7, 10)). There is also evidence for activation of the UPR in human GN and DN (7, 10). In general, the UPR is beneficial, as it promotes protein folding thereby alleviating injury, although severe/prolonged ER stress can be detrimental and can lead to apoptosis. Autophagy is another cytoprotective process that helps clear misfolded proteins from cells, and it may be linked to the UPR (7, 11). There is considerable evidence that the UPR and autophagy contribute to the maintenance of podocyte/glomerular homeostasis under basal conditions and that they attenuate disease (7, 10). For example, mice with podocyte-specific genetic deletion of IRE1α show reduced induction of ER chaperones and autophagy that is associated with podocyte injury as the mice age, and exacerbation of injury in experimental glomerulopathies. “Preconditioning” of animals to induce the UPR or treatment of animals with chemical chaperones to reduce ER protein misfolding attenuate glomerular disease (7, 12, 13). Mice with podocyte-specific deletion of the key autophagy mediators ATG5 or ATG7 show age-related podocyte injury and exaggerated injury in experimental glomerulopathies (7, 14). Together, these observations support mechanistic roles for the UPR and autophagy in glomerular health and disease, particularly in attenuating injury or promoting repair. Furthermore, there is recent evidence that stimulating IRE1α and the UPR pharmacologically may protect cells from injury induced by protein misfolding (15).

While multiple mechanisms mediate the pathogenesis of GN and DN, defining specific pathways that mediate injury, such as ER stress/UPR and autophagy, may be an opportunity for the establishment of more reliable diagnostic testing and development of precise therapies. In this study, we examined gene transcriptional pathways in human GN and DN, and we provide evidence that MN, FSGS, IgAN and DN are all associated with increased expression of ER/UPR and autophagy genes. We then used this knowledge of gene expression in glomerulopathies to search for drugs that activate analogous gene expression pathways in cells, with the intent of selecting candidate drugs for mechanism-based therapeutics. Using this approach, we identified neratinib as a drug that ameliorated injury in cultured GECs.

## Materials and methods

### Dataset analyses

The publicly accessible Nephroseq dataset “JuCKD-Glom” (GSE47183) was used for the expression analysis of glomerular genes, including genes associated with the ER/UPR and autophagy (16, 17). Nephroseq contains microarray gene expression data (mRNAs) of laser-captured glomeruli from human kidney biopsies. These data are presented as the fold-increase of gene expression in disease above healthy control. Additional analyses were performed using publicly accessible datasets GSE108109 (18), GSE115857 (unpublished) and GSE141295 (19) (Supplementary Table S1). It should be noted that since these datasets have been generated/published independently, we did not pool the data together and we analyzed the datasets separately. The *p*-values and p-values adjusted for the false discovery rate (e.g., Q-values), which we present in this manuscript, are those calculated and reported within the datasets by their original authors. We employed principal component analysis of gene expression, an algorithm that identifies the maximal variations in the data and reduces the dimensionality to a few components (17, 20). Pathway overrepresentation and gene ontology (GO) enrichment analyses were performed using the ConsensusPathDB interaction database (21, 22). This database provides the adjusted *p*-values (hypergeometric test, corrected for multiple comparisons) that we present in this manuscript (22).

To search for gene signatures induced by chemical perturbagens (drugs), i.e., “connectivity mapping,” as well as ligands and protein kinases, we used the Library of Integrated Network-based Cellular Signatures (LINCS) L1000 dataset (23, 24), which is an extension of the original connectivity mapping (CMAP) dataset (“old CMAP”) (25), and contains 1,319,138 profiles from 42,080 perturbagens (19,811 small molecules, 18,493 shRNAs, 3,462 cDNAs and 314 biologics; treatment vs. vehicle control pairs), corresponding to 25,200 biological entities and 473,647 signatures carried out in 3–77 cells lines. To query this database, we utilized LINCS analytical tools,^1^ including BD2K-LINCS DCIC and Enrichr. Signatures from the LINCS L1000 data are computed using the moderated Z-score method; the *p*-values and adjusted *p*-values presented in this manuscript are those reported directly by the LINCS data portal. One limitation of this dataset is that it includes the HA1E immortalized normal kidney epithelial cell line and various tumor cell lines, but glomerular cells are not included.

Relationships between GO terms were produced by using the QuickGO tool (26). The chord plot was produced by using the R package tool GOPlot (27). Protein–protein interaction networks were constructed using NetworkAnalyst^2^ and STRING interactome database with medium confidence score of 600 and experimentally verified criteria (28).

### Generation of ER/UPR and autophagy gene lists

To compile a set of genes associated with ER function and ER stress/UPR, we combined genes listed in the Protein Processing in the ER KEGG pathway (which includes UPR and other ER-related genes) (29), and in the Qiagen human UPR PCR Array (PAHS-089Z, Qiagen) (Supplementary Table S2). Second, we examined genes or genes encoding proteins that were reported to be inducible by X-box binding protein-1 (XBP1; an effector of IRE1α), ATF6 and XBP1 + ATF6 in HEK293T cells (30). There was a total of 639 genes in this dataset, of which 70 were also present in the KEGG+Qiagen dataset (Supplementary Table S2). We then subjected the non-overlapping 570 XBP1- and ATF6-inducible genes to a GO analysis (Supplementary Table S3), and we selected genes corresponding to ER/UPR pathways. This resulted in an additional 116 genes, which we added to the 203 ER/UPR genes in the KEGG+Qiagen dataset for a total of 319 ER/UPR genes (Supplementary Table S4). Of the 319 ER/UPR genes, 271 (85%) were found in the Nephroseq microarray (48 were not in the microarray).

To compile a set of autophagy-related genes, we took a list of 98 genes from the Autophagy KEGG pathway and Qiagen autophagy PCR array (PAXX-084Y, Qiagen), and we subjected these genes to a GO analysis (Supplementary Table S5). Then, we selected autophagy-related pathways and genes corresponding to these GO pathways. This resulted in an additional 449 genes, which we added to the original 98 genes for a total of 547 autophagy genes (Supplementary Table S4). Of the 547 autophagy genes, 391 (71%) were found in the Nephroseq microarray (156 were not in the microarray). Protein–protein interaction networks were produced based on proteins encoded by the 319 ER/UPR and 547 autophagy genes. The large majority of the ER/UPR (Supplementary Figure S1a) and autophagy genes (Supplementary Figure S1b) were mapped to these networks, and the majority of these genes interconnected with each other. There was also a small number of intermediate proteins that connected the selected ER/UPR and autophagy genes (Supplementary Figure S1). As expected, the largest nodes in the ER/UPR network are chaperones and ubiquitin-proteasome system components.

### GEC culture, immunoblotting and quantification of FACs

Primary mouse GECs were generated according to previously published methods (12, 31). These cells contain a floxed IRE1α gene, but in the absence of transduced Cre recombinase, the cells express normal levels of IRE1α, and are phenotypically normal (12). In IRE1α knockout (KO) cells, Cre recombinase has deleted IRE1α (12). Studies were done after the cells were cultured at the differentiation temperature (37°C). In experiments, GECs were incubated with drugs added to culture medium. Drugs included adriamycin (doxorubicin), chloroquine, tunicamycin (Sigma-Aldrich Canada, Oakville, ON); geldanamycin, radicicol, NVP-AUY922 (luminespib), neratinib (Cayman Chemical, Ann Arbor, MI); rapamycin (BioShop Canada, Burlington, ON); or IXA6 (N-[(4-chlorophenyl)methyl]-N-[2-(2,3-dihydro-1H-indol-1-yl)-2-oxoethyl]pyridine-3-sulfonamide; Life Chemicals Inc., Niagara-on-the-Lake, ON). Stock solutions of drugs were prepared in DMSO.

The protocol for immunoblotting was described previously (12). Antibodies included rat anti-GRP94 (sc-32,249; Santa Cruz Biotechnology, Santa Cruz, CA), rabbit anti-BiP/GRP78 (ADI-SPA-826F, Enzo Life Sciences, Ann Arbor, MI), mouse anti-HSP70 (3A3, sc-32,239, Santa Cruz), rabbit anti-LC3B (2,775, Cell Signaling Technology, Danvers, MA), rabbit anti-p62/SQSTM1 (23,214, Cell Signaling), rabbit anti-mesencephalic astrocyte-derived neurotrophic factor (MANF/ARMET; PAB13301, Abnova, Walnut, CA), and rabbit anti-actin (A2066, MilliporeSigma, Mississauga, ON). Chemiluminescence signals were detected in a ChemiDoc Touch Imaging System (Bio-Rad; Mississauga, ON). The intensities of bands in each immunoblot were measured in samples derived from the same experiment and were quantified using ImageJ software. The actin signal was used as loading control for normalization of signals. We then calculated the relative intensities of all bands in each immunoblot. We ensured that the intensities of signals were all within a linear range. The lactate dehydrogenase (LDH) release assay was described previously (31).

To visualize FACs, cultured GECs were fixed with 4% paraformaldehyde (37°C), permeabilized with 0.5% Triton X-100 and blocked with 3% BSA. Cells were stained with mouse antibody against the FAC adaptor protein vinculin (Sigma V9131) for 24 h (4°C) plus rhodamine-goat-anti-mouse IgG, as well as fluoresceinated-phalloidin (to stain F-actin and outline the cells), and Hoechst H33342 (to stain nuclei), as described previously (17, 32). Z-stack images were acquired on a Zeiss Axio Observer inverted fluorescence microscope with visual output connected to an AxioCam MRm monochrome camera (Carl Zeiss AG; Toronto, ON). Immunofluorescence intensities and cell measurements were performed using ImageJ, as described previously (17, 32). ImageJ allows pre-definition of particle size and the threshold of immunofluorescence intensity to count FACs and estimate cell size.

### Statistics

As noted above, the statistical parameters (*p*-values and adjusted p-values) of gene expression datasets, as well as the ConsensusPathDB interaction and L1000 databases that we present in this manuscript are those calculated and reported within these datasets. To address the overlap of genes among GNs, we calculated Jaccard similarity coefficients and assessed the significance using a hypergeometric test. Experimental data that we generated are presented as mean ± standard deviation (SD) or standard error (SE; as indicated). Comparisons between two groups were done by a two-tailed Student’s t-test. For three or more groups, statistical differences were assessed using one-way analysis of variance. Where significant differences were found, post-hoc analyses were performed using Sidak’s multiple comparisons test.

## Results

### Human glomerulopathies reveal many shared upregulated genes

**Figure 1:**
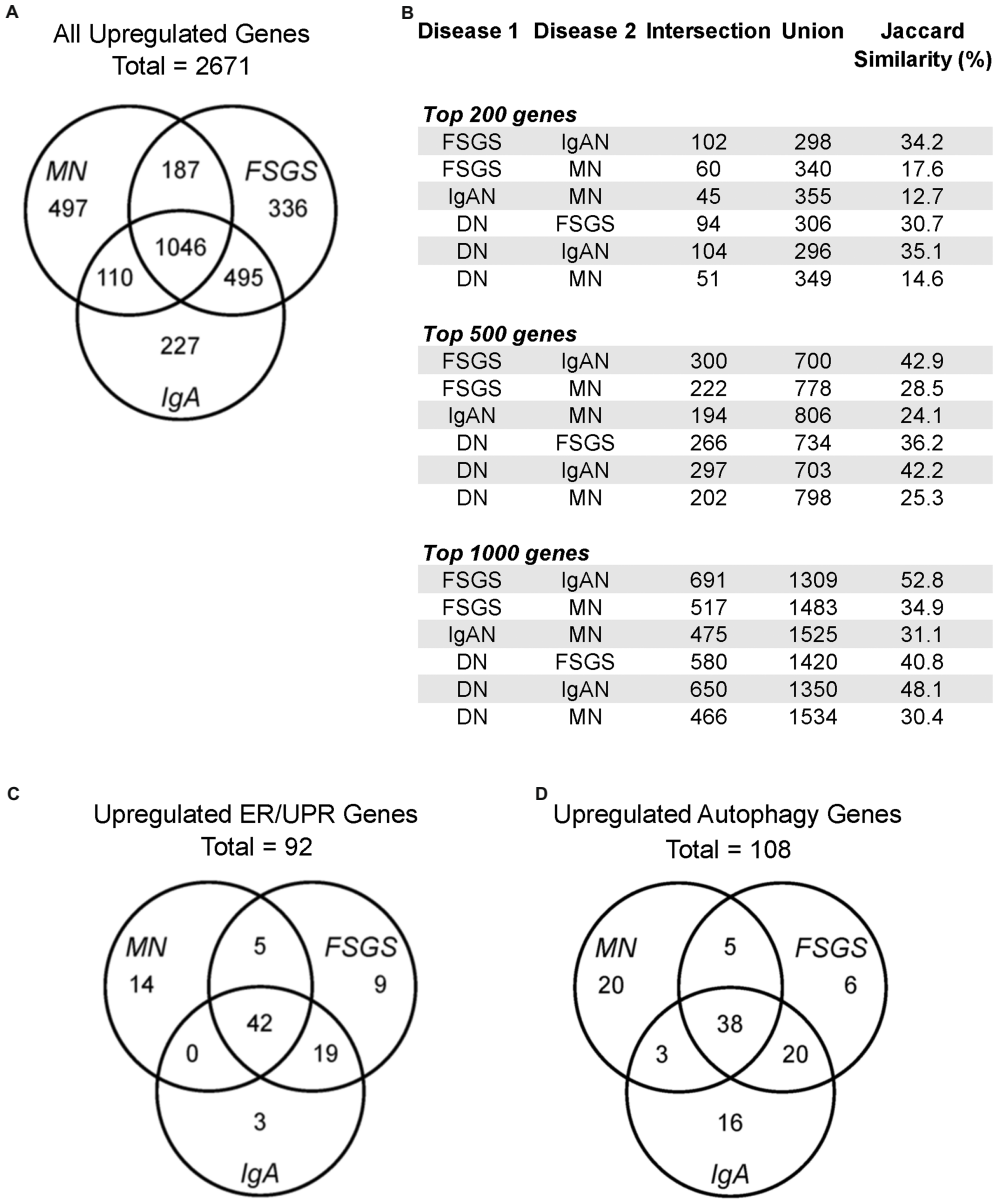
Gene expression in human glomerulopathies. **(A,C,D)** Venn diagrams show numbers of upregulated genes, including all genes, ER/UPR genes and autophagy genes in the JuCKD-Glom Nephroseq database. **(B)** Jaccard similarities of genes upregulated in glomerulopathies. All overlaps (intersections) are statistically significant; *p* < 1 × 10^−37^ (hypergeometric test).

The datasets employed and the workflow for this study are presented in Table 1. Initially, we examined all upregulated genes in FSGS (*N* = 25), MN (*N* = 21) and IgAN (*N* = 27) in the JuCKD-Glom (GSE47183) dataset in Nephroseq (Supplementary Table S1) (16). This dataset contains microarray gene expression data of laser-captured glomeruli from human kidney biopsies. The dataset provides the fold-change in gene expression compared with healthy controls (*N* = 21). The mean ages of the patients with the three GNs ranged from 36.4 to 53.7 years, compared with 47.2 years in controls (Supplementary Table S1). The mean estimated glomerular filtration rates of the three GNs were slightly reduced (73.9–74.5 ml/min/1.73m^2^), compared with control (105.4 ml/min/1.73m^2^), while blood pressures were in the normal range (Supplementary Table S1). A total of 2,671 genes (out of 11,933 examined) showed increased expression in the three GNs; MN = 1,840, FSGS = 2,064 and IgAN = 1,878 (Figure 1A; Supplementary Table S6). There were 1,046 upregulated genes common to the three GNs, i.e., more than 50% of genes upregulated in each GN were common to the three (Figure 1A). Furthermore, a number of additional genes were common to at least two GNs (Figure 1A). We calculated the Jaccard similarity coefficients corresponding to the overlap of the top 200, 500 and 1,000 upregulated genes in the GNs (Figure 1B). The results suggested a highly significant overlap (hypergeometric test *p* < 1 × 10^−37^ in all cases), confirming that upregulated genes were shared among diseases.

**Table 1:** Datasets and workflow for the study.

**Figure 2:**
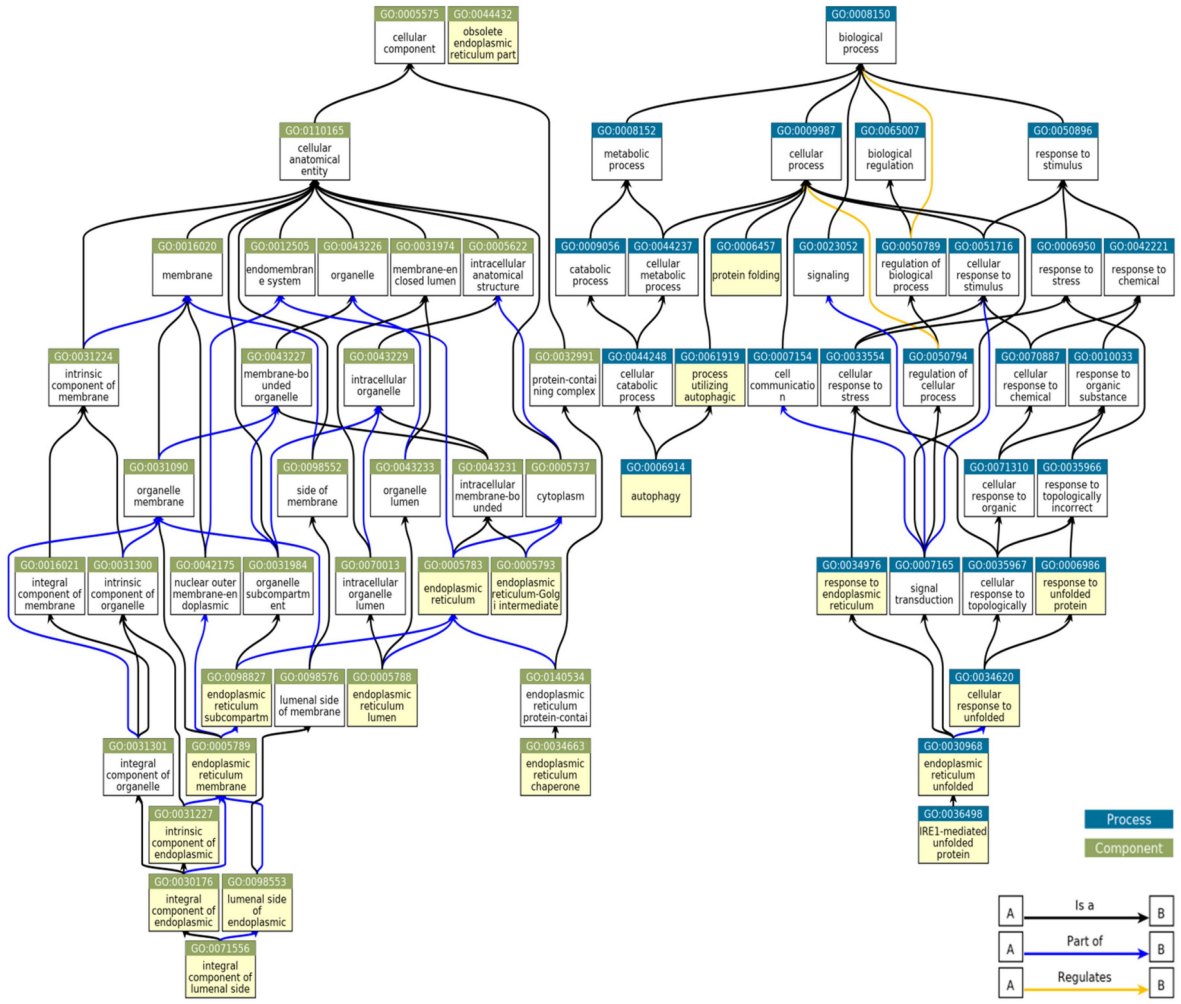
Relationships between GO terms selected from genes upregulated in MN, FSGS, and IgAN JuCKD-Glom datasets. Boxes highlighted in yellow denote 19 GO terms represented in the glomerulopathies. “A is a B”: node A is a subtype of node B; “A part of B”: node A is a part of node B; “A regulates B”: node A regulates node B positively or negatively.

**Figure 3:**
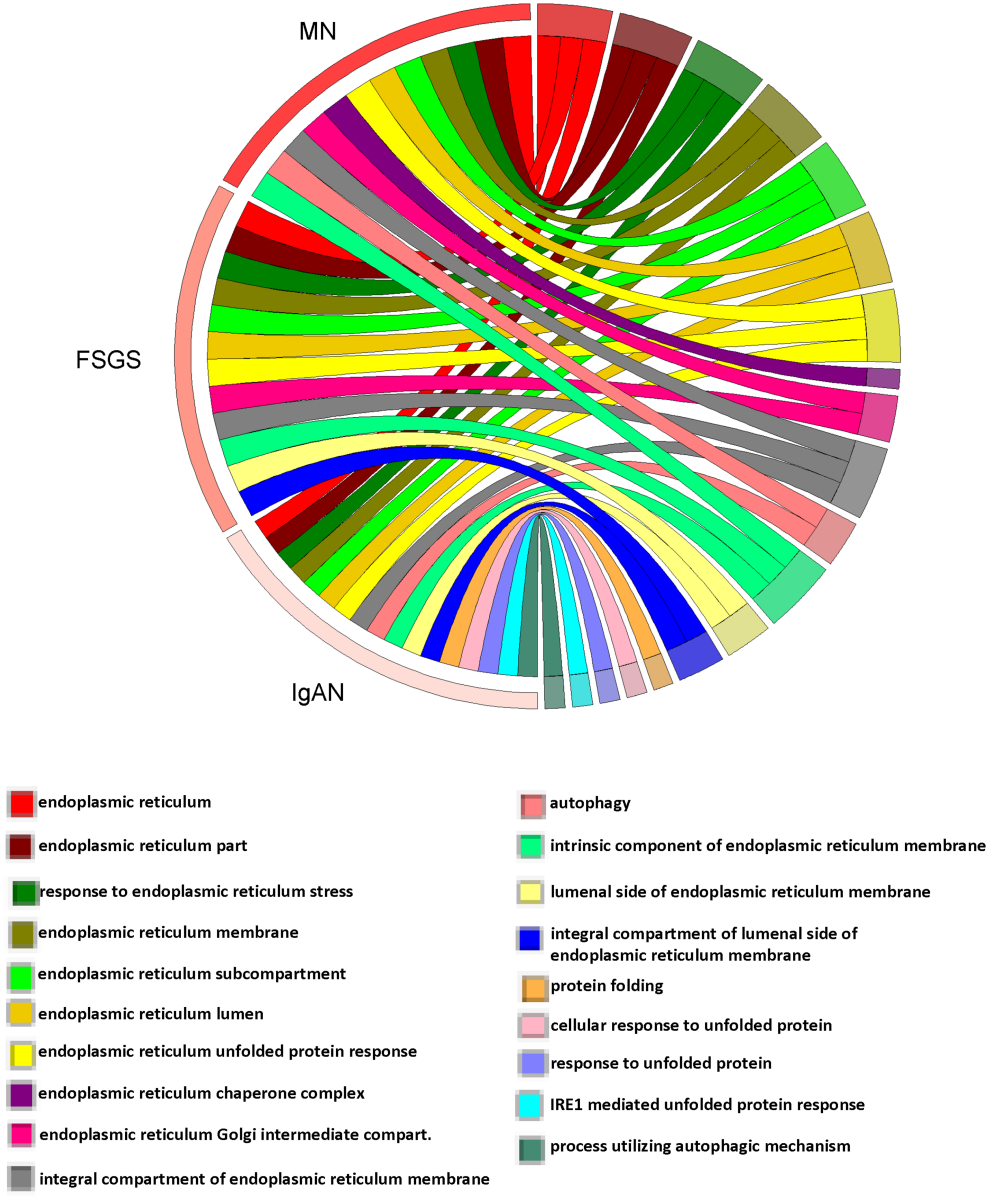
Occurrence summary (chord plot) between 19 GO terms selected from genes upregulated in MN, FSGS, and IgAN JuCKD-Glom datasets (highlighted in yellow in Figure 2). The figure highlights pathways common among the three glomerulopathies.

**Figure 4:**
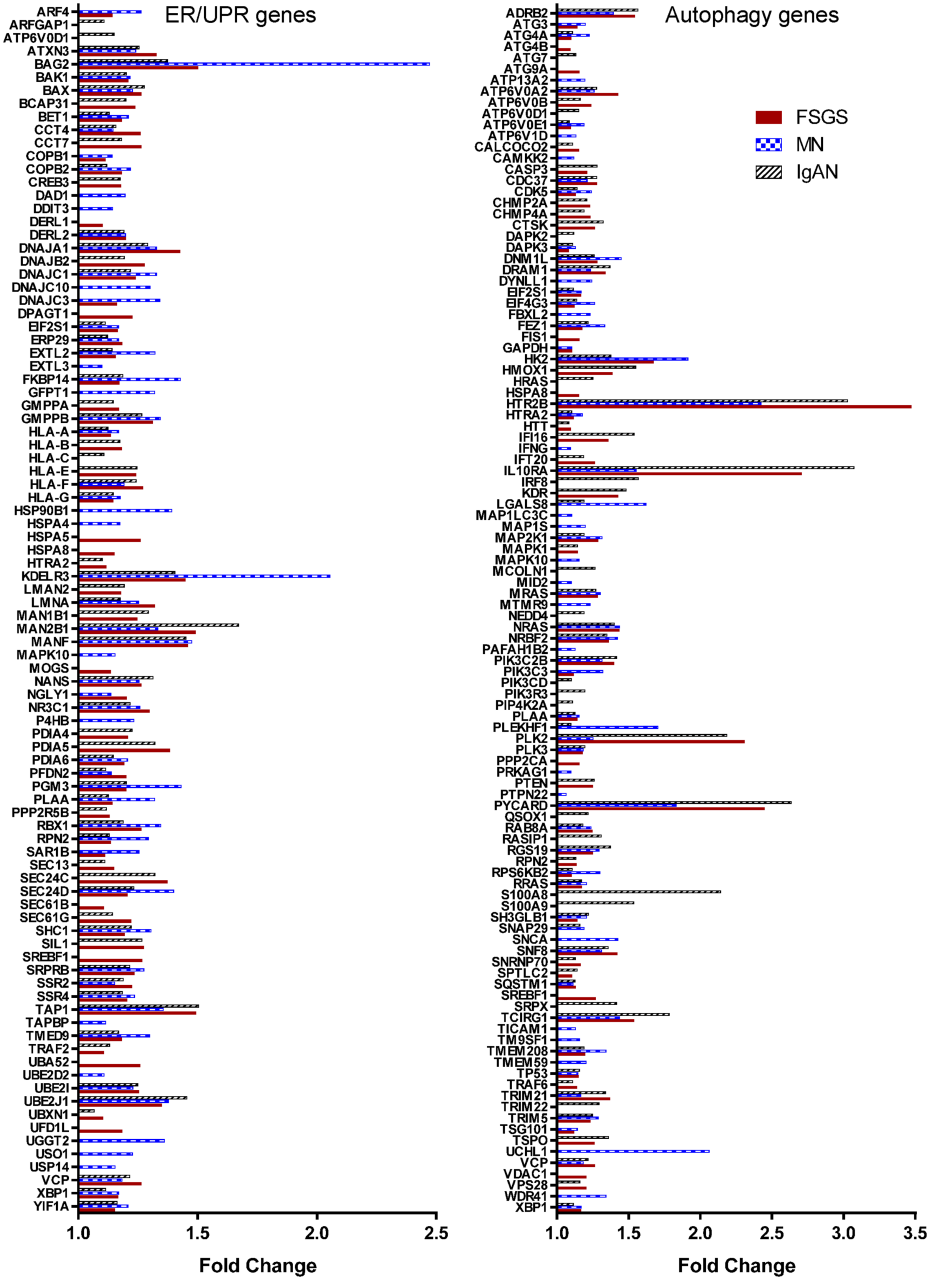
ER and autophagy genes that are upregulated significantly in glomerulopathies. The figure presents the 93 ER/UPR genes and 108 autophagy genes whose expression was increased in at least one the three glomerulopathies.

**Figure 5:**
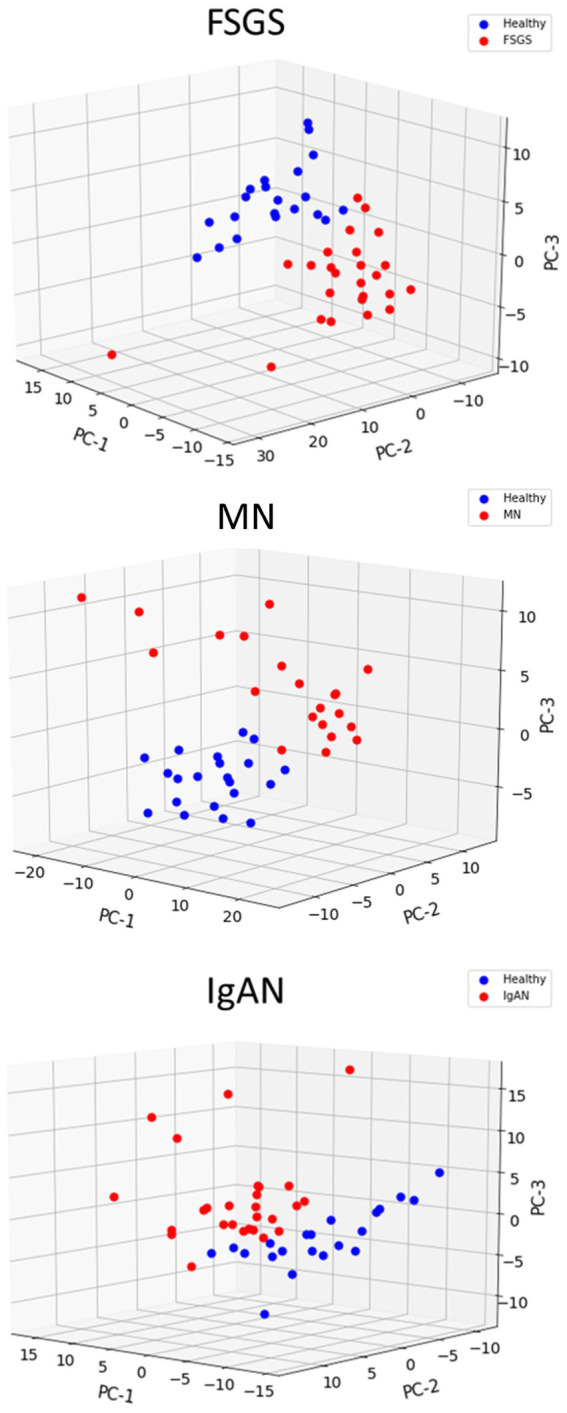
Principal component analysis of ER/UPR gene expression in glomerulopathies. Healthy controls are clearly separated from patients with FSGS, MN and IgAN.

**Figure 6:**
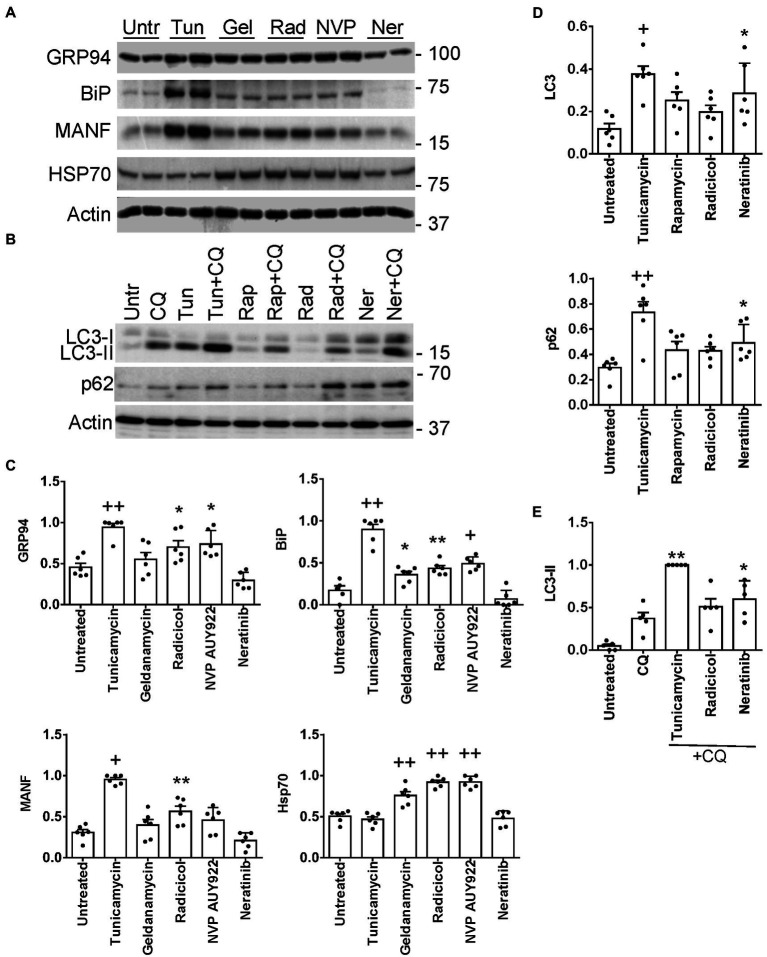
Induction of UPR **(A,C)** and autophagy genes **(B,D)** by L1000-ER/UPR/autophagy drugs. Cultured GECs were incubated for 24 h with drugs (10 μM concentrations). In panel **B**, drugs were added together with CQ, as indicated. Lysates were immunoblotted, as indicated. **(A,B)** Representative immunoblots. **(C–E)** Densitometric quantification (the expression of each protein is normalized to actin). *p < 0.05, **p < 0.01, ^+^*p* < 0.001, ^++^*p* < 0.0001 vs. untreated. Mean ± SE, 3 experiments performed in duplicate. **(E)** Autophagosomal flux, as determined by the level of LC3-II in the presence of chloroquine (densitometric quantification of specific lanes in panel **B**). *p < 0.05, **p < 0.01, vs. chloroquine. Tunicamycin and rapamycin are stimulators of the UPR or autophagy (positive controls). Untr, untreated; Tunic, tunicamycin, Gel, geldanamycin; Rad, radicicol; NVP, NVP-AUY922; Ner, neratinib; Rap, rapamycin; CQ, chloroquine.

**Figure 7:**
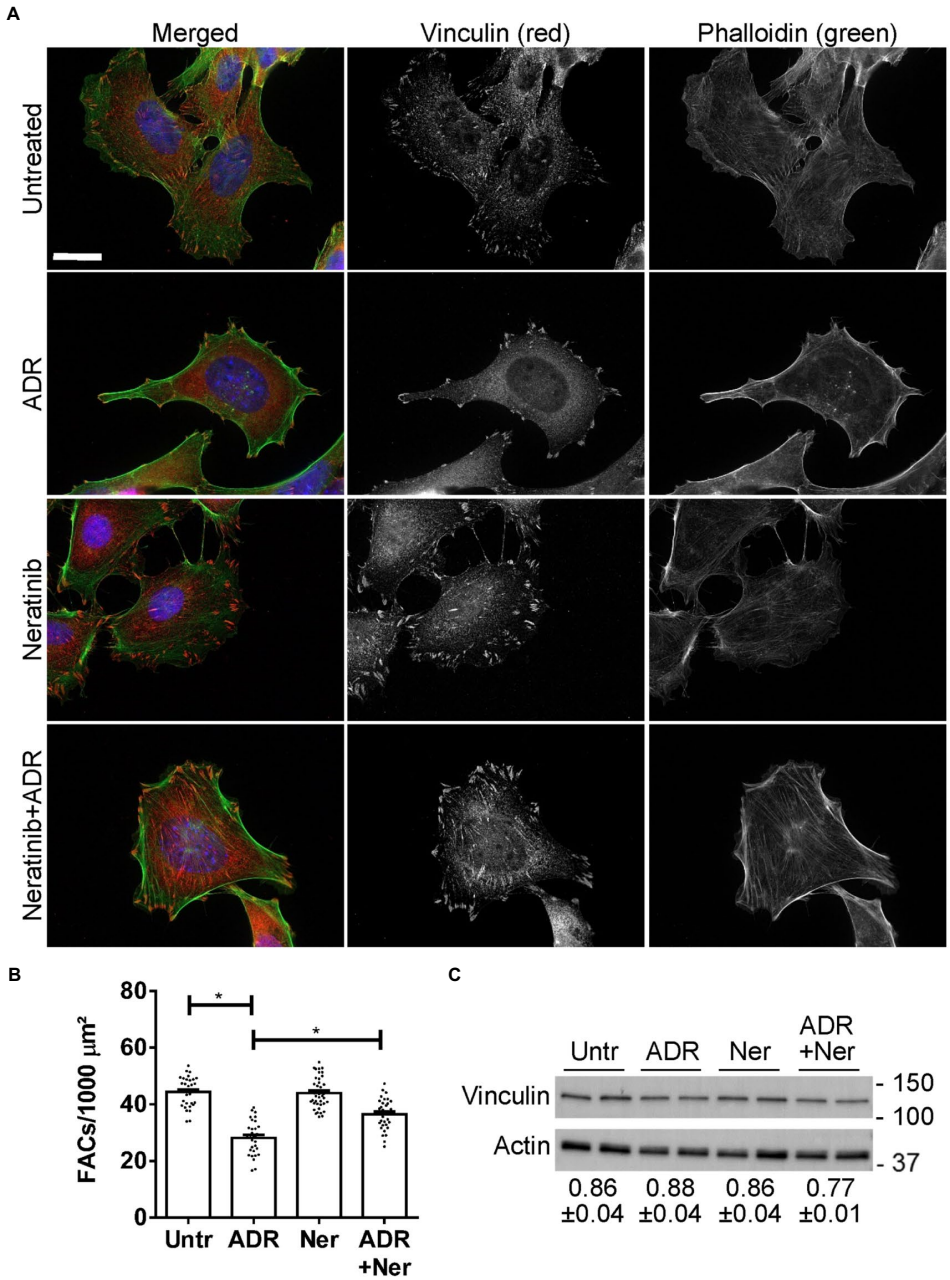
Effect of neratinib on FACs. Cultured GECs were untreated, or were incubated with adriamycin (ADR; 0.5 μM) together with or without neratinib (Ner; 100 nM) for 24 h. **(A** and **B)** Cells were then stained with anti-vinculin antibody (red; FACs), phalloidin (green; F-actin cytoskeleton) and H33342 (blue; nuclei). Representative photomicrographs **(A)** and quantification of FACs **(B)** are presented. FACs are evident throughout the cells, and F-actin bundles are seen to originate within the FACs. ADR reduces FACs and this effect is attenuated by neratinib. Bar = 25 μm. *p < 0.0001 Untreated vs. ADR and ADR vs. ADR + neratinib. Mean ± SE, 3 experiments with 10–16 measurements per group per experiment. **(C)** Representative immunoblot of vinculin shows comparable levels among the four conditions. Densitometric quantification of 4 experiments performed in duplicate is shown below the blot (vinculin normalized to actin; mean ± SE). There are no statistically significant differences.

**Figure 8:**
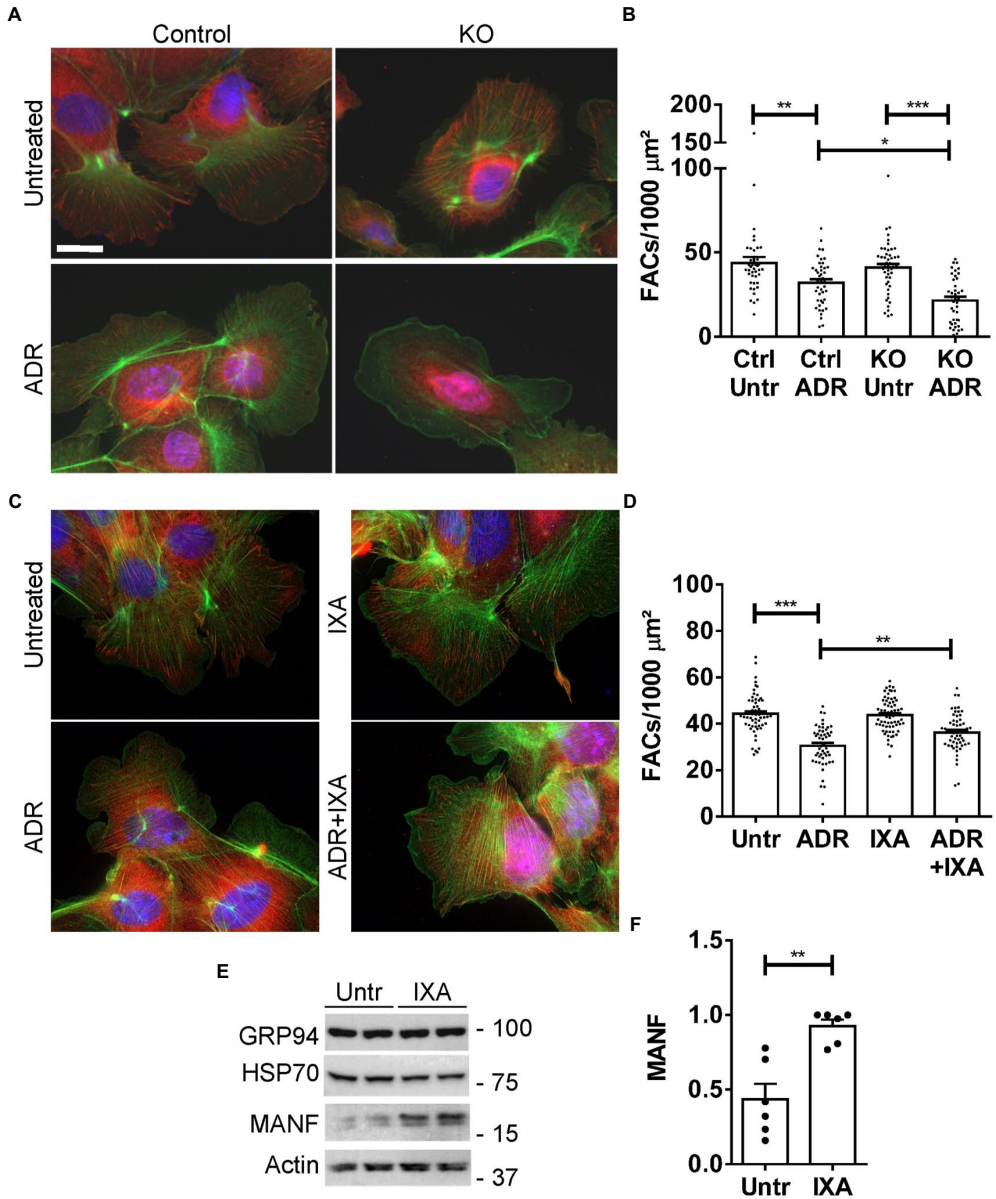
Effect of the UPR and IXA6 on FACs. **(A,B)** Control and IRE1α KO GECs were untreated or were incubated with adriamycin (ADR; 0.5 μM) for 24 h. Cells were then stained with anti-vinculin antibody (red; FACs), phalloidin (green; F-actin cytoskeleton) and H33342 (blue; nuclei). Representative photomicrographs **(A)** and quantification of FACs **(B)** are presented. The number of FACs was reduced after ADR, and the reduction was greater in IRE1α KO cells compared to control. Bar = 25 μm. *p < 0.05, **p < 0.01, ***p < 0.0001. Mean ± SE, 3 experiments with 10–20 measurements per group per experiment. **(C** and **D)** GECs were untreated, or were incubated with ADR together with or without IXA6 (IXA; 10 μM) for 24 h. Cells were then stained as above. IXA6 attenuated the reduction in FACs induced by ADR. **p < 0.01, ***p < 0.0001. Mean ± SE, 4 experiments with 10–21 measurements per group per experiment. **(E,F)** GECs were incubated with IXA6 (10 μM) for 24 h. Lysates were immunoblotted as indicated. Representative immunoblots **(E)** and densitometric quantification of MANF normalized to actin **(F)** are presented **p < 0.01. Mean ± SE, 3 experiments performed in duplicate.

Part 1: Datasets employed (publicly accessible) and those compiled
(1) Datasets of glomerular gene expression
(a) Primary dataset: Nephroseq dataset “JuCKD-Glom” (GSE47183)
(b) Confirmatory datasets: GSE108109, GSE115857 and GSE141295
(*Supplementary Table S1*)
(2) Compilation of a set of genes associated with ER function and ER stress/UPR
(a) ER KEGG pathway (which includes UPR and other ER-related genes)
(b) Qiagen human UPR PCR Array (PAHS-089Z, Qiagen)
(c) XBP1- and ATF6-inducible genes subjected to a GO analysis, and selection of genes corresponding to ER/UPR pathways
(d) Mapping protein–protein interaction networks (based on above genes)
(*Supplementary Tables S2–S4*; *Supplementary Figure S1a*)
(3) Compilation of a set of autophagy-related genes
(a) Autophagy KEGG pathway
(b) Qiagen autophagy PCR array (PAXX-084Y, Qiagen)
(c) Above genes were subjected to a GO analysis (*Supplementary Table S1*), and then autophagy-related pathways and genes corresponding to these GO pathways were selected
(d) Mapping protein–protein interaction networks (based on above genes)
(*Supplementary Tables S4–S5*; *Supplementary Figure S1b*)
(4) Dataset for Connectivity Mapping (CMAP)
Library of Integrated Network-based Cellular Signatures (LINCS) L1000 dataset
(An extension of the “old CMAP” dataset, containing profiles of perturbagens, including drugs)
Part 2: Workflow for glomerular gene expression analysis
(1) Analysis of all upregulated genes in human glomerulopathies in the JuCKD-Glom (GSE47183) dataset in Nephroseq
(*Figure 1*; *Supplementary Table S6*)
(2) Pathway overrepresentation and gene ontology enrichment analyses of above genes to determine if there is activation of pathways associated with ER stress/UPR and autophagy
(*Figures 2*, *3*; *Supplementary Table S7–S8*)
(3) Analysis of all downregulated genes in human glomerulopathies
(*Supplementary Table S9*)
(4) ER and autophagy gene expression in human glomerulopathies (JuCKD-Glom)
a) Define the ER/UPR and autophagy genes whose expression is increased in glomerulopathies (using compiled sets of genes associated with ER function and ER stress/UPR, as well as autophagy; from Part 1)
(*Figures 1*,*4*; *Supplementary Table S10*)
(b) Principal component analysis of ER/UPR gene expression in glomerulopathies
(*Figure 5*)
(5) Validation of upregulated ER/UPR and autophagy genes using additional glomerular datasets (GSE108109, GSE115857 and GSE141295)
(*Supplementary Table S1*)
(6) Examination of ER/UPR and autophagy gene expression by glomerular cell type
(*Supplementary Table S11*)
Part 3: Workflow for connectivity mapping (to identify drugs)
(1) Using the LINCS L1000 dataset, search for drugs (“chemical perturbagens”) which produce gene expression signatures in cell lines that show a statistically significant match with the upregulated ER/UPR and autophagy genes in glomerulopathies (based on JuCKD-glom dataset)
(*Supplementary Tables S12–S14*)
(2) Selection of six “L1000-ER/UPR/autophagy drugs” that show matches with both the ER/UPR and autophagy gene signatures and for which descriptions are available in the literature
(*Supplementary Table S12*)
(3) Validation of the selected drugs using additional glomerular datasets (GSE108109, GSE115857 and GSE141295)
(*Supplementary Table S13*)
Part 4: Experimental verification of drug effects
(1) Verification that L1000-ER/UPR/autophagy drugs stimulate expression of ER chaperones and/or proteins involved in autophagy (in GECs)
(*Figure 6*)
(2) Verification that the L1000-ER/UPR/autophagy drug neratinib reduces GEC injury
(a) Lactate dehydrogenase assay
(*Supplementary Table S12*)
(b) Focal adhesion complex (FAC) assay
(*Figure 7*)
(3) Demonstration that IXA6, a drug directed at IRE1α RNase, reduces GEC injury
(a) Verification that the UPR protects GECs from injury in the FAC assay
(b) Validation that IXA6 attenuates GEC injury
(*Figure 8*)

The major components of the study are presented in four parts, with each part divided into sub-sections. The tables and figures showing the results are indicated at the bottom of each sub-section (italics).

To determine if among the upregulated genes in MN, FSGS and IgAN there were genes associated with ER stress and the UPR, as well as autophagy, we performed a GO enrichment analysis (Supplementary Table S7). Examination of all GO terms in each GN revealed a number of GO terms associated with ER components, ER function and the UPR, such as ER chaperones, protein folding, protein transport, stress responses and others (Figure 2; Supplementary Table S8). A smaller number of GO terms were associated with autophagy (Figure 2; Supplementary Table S8). Approximately 50% of 19 selected GO terms were present in all three GNs with a further 20% in two (Figure 3). Together, these results imply that the UPR and autophagy are common responses in multiple types of glomerular injury.

In JuCKD-Glom, there are also genes whose expression is downregulated in GNs (Supplementary Table S9). We performed a GO analysis combining all downregulated genes that were common to at least two of MN, FSGS and IgAN (*N* = 2,257). No ER/UPR or autophagy pathways were found and there were only 3 ER-related ontology terms (Supplementary Table S9). Probably, downregulation of UPR and autophagy genes is less biologically relevant, compared with upregulation, and since our focus is on the UPR and autophagy, we do not include further analysis of downregulated genes in the present study.

### ER and autophagy gene expression is increased in human glomerulopathies

We compiled a set of 319 genes associated with ER function/stress/UPR (ER/UPR) and a second set of 547 genes associated with autophagy (Materials and methods). Among these genes, 271 and 391, respectively, were present in the Nephroseq microarray. FSGS, MN and IgAN all demonstrated increases in the expression of ER/UPR genes. Among 271 ER/UPR genes, 92 were increased when the three GNs were considered together, and of these, 42 were common to the three diseases (Figures 1C, 4; Supplementary Table S10). Furthermore, a significant number of ER/UPR genes were common to at least two GNs (Figure 1C; Supplementary Table S10). Principal component analysis of changes in ER/UPR gene expression indicates that normal controls and FSGS, MN and IgAN patients can be clearly separated into non-overlapping populations (Figure 5). By analogy, FSGS, MN and IgAN all demonstrated increases in the expression of autophagy genes. Among 391 autophagy genes, 108 were increased when the three GNs were considered together, and of these 38 were common to the three diseases (Figure 1D, 4; Supplementary Table S10). Similar to the ER/UPR genes, a significant number of autophagy genes were common to at least two GNs (Figure 1D; Supplementary Table S10).

The JuCKD-Glom dataset also contains microarray data of laser-captured glomeruli from patients with DN. Although DN is not a primary GN, we nevertheless examined expression of genes in DN, given its clinical importance. The Jaccard similarity coefficients and the highly significant *p*-values (hypergeometric test *p* < 1 × 10^−37^ in all cases) of the overlap of the top upregulated genes in DN and the GNs indicated that upregulated genes were common not only among FSGS, MN and IgAN, but also among DN and the three GNs (Figure 1B). Moreover, there were 34 ER/UPR genes and 44 autophagy genes upregulated in DN. Interestingly, there was only one ER/UPR gene (Supplementary Table S10) and two autophagy genes (Supplementary Table S10) upregulated in DN, which were not part of the groups of genes upregulated in FSGS, MN and IgAN. These genes were added to the 92 ER/UPR and 108 autophagy genes for a total of 93 ER/UPR and 110 autophagy genes, which were used in further studies. Of the 93 upregulated ER/UPR genes and 110 upregulated autophagy genes, 89 were mapped to the ER/UPR protein–protein interaction network, and 106 to the autophagy network, respectively (Supplementary Figure S1).

### Upregulation of ER and autophagy genes is recapitulated in additional GN datasets

To determine if upregulation of genes in the JuCKD-Glom GN dataset (GSE47183) was more broadly applicable, we studied gene expression in additional datasets of GN and control subjects, including GSE108109, GSE115857 and GSE141295 (Supplementary Table S1) (18, 19). We selected these datasets because there were at least 10 human glomerular GN samples per dataset, each dataset provides the fold-change in gene expression compared with healthy controls, and the samples were ascertained to be distinct from those in JuCKD-Glom. When examining upregulation of individual genes (i.e., the 319 ER/UPR genes and 547 autophagy genes), at least as many of these genes were upregulated in GSE108109, GSE115857 and GSE141295 as in JuCKD-Glom, although only a modest to moderate number of these genes overlapped with those observed in JuCKD-Glom (Supplementary Table S1). This may be at least in part due to the use of different microarrays or techniques between the datasets. Importantly, a substantial majority of the ER/UPR and autophagy-related GO biological pathways and gene categories that were evident in JuCKD-Glom overlapped with those identified in GSE108109, GSE115857 and GSE141295, and there were additional biological pathways and gene categories related to the ER/UPR and autophagy identified in these datasets (Supplementary Table S8). Together, these analyses of gene expression support the view that ER/UPR and autophagy pathways are activated in human GNs.

### ER/UPR and autophagy genes are expressed in multiple glomerular cell types

To determine if the upregulated ER/UPR (*N* = 93) and autophagy genes (*N* = 110) in glomerulopathies (Figure 4) were expressed in a specific glomerular cell type, we interrogated single cell RNA sequence datasets. In two older datasets that examined a limited number of glomerular cells (33, 34), 73/93 ER/UPR genes and 63/110 autophagy genes were expressed in podocytes, while 70/93 ER/UPR genes and 74/110 autophagy genes were expressed in glomerular mesangial cells (Supplementary Table S11). In a more recent and more extensive dataset (35), 86/93 ER/UPR genes and 100/110 autophagy genes were expressed in podocytes, and the same genes were expressed in glomerular endothelial cells (Supplementary Table S11). It is therefore reasonable to conclude that at least basal expression of these genes is ubiquitous and is not restricted to a single glomerular cell type.

### Connectivity mapping (CMAP) identifies drugs that target the ER/UPR and autophagy

MN, FSGS and IgAN showed common signatures in UPR and autophagy gene expression (Figure 1), as well as biological pathways and gene categories (Supplementary Table S8). We proceeded to use the LINCS L1000 dataset to identify candidate drugs for treatment of glomerulopathies by relating the gene expression signatures of different drugs with the 93 ER/UPR and 110 autophagy genes upregulated in these glomerulopathies (JuCKD-Glom). A considerable number of studies (see Introduction) indicate that the UPR and autophagy are cytoprotective mechanisms in glomerular diseases, and this cytoprotection is believed to be dependent on upregulation of UPR and autophagy genes/proteins (7, 10). Therefore, in contrast to other studies that have evaluated drugs as inhibitors, we asked which drugs could potentially stimulate the UPR or autophagy, i.e., induce upregulated gene signatures in cultured cell lines that are *similar* to the upregulated gene signatures in glomerular diseases? This approach is supported by recent studies that have demonstrated cytoprotective effects of drugs that *stimulate* the IRE1α-XBP1 UPR pathway (15, 36).

Given that the glomerulopathies have a high degree of overlap in gene expression profile, we used the *union* of the upregulated genes in the connectivity mapping analysis. The union allows use of a greater number of genes in the analysis and to identify candidate drugs with greater statistical power. In addition to drugs, we searched for signatures induced by ligands (growth factors, cytokines) and protein kinase knockdowns with shRNAs. In the LINCS L1000 dataset, there were 52 drugs (“chemical perturbagens”) in 17 cell lines (Supplementary Tables S12, S13), which produced gene signatures that showed a statistically significant similarity (adjusted *p* < 0.05) with the 93 upregulated ER/UPR genes in glomerulopathies (Supplementary Table S10). By analogy, there were 227 drugs in 27 cell lines (Supplementary Tables S12, S13), which produced gene signatures showing significant similarity with the 110 upregulated autophagy genes in glomerulopathies (Supplementary Table S10). Among these two groups of drugs, there were six drugs that showed similarities with *both* the ER/UPR and autophagy gene signatures, and for which descriptions were available in the literature (Supplementary Table S12). We selected these six candidate drugs for further characterization and refer to them as “L1000-ER/UPR/autophagy drugs.” Within this group, geldanamycin, NVP-AUY922 (luminespib) and radicicol are Hsp90 inhibitors, although radicicol may have multiple actions. Hsp90 inhibition can lead to the activation of cytosolic and ER stress responses. Neratinib is a highly selective epidermal growth factor receptor (EGFR) and HER2 inhibitor. Celastrol and withaferin-a have multiple reported actions, including activation of cytosolic and ER stress responses (37, 38). Connectivity mapping of the UPR genes *common* to MN, FSGS and IgAN demonstrated the presence of the same six L1000-ER/UPR/autophagy drugs (Supplementary Table S13), in keeping with our approach that used the *union* of the upregulated genes.

Comparison of ER/UPR gene signatures induced by the L1000-ER/UPR/autophagy drugs showed that the drugs with effects in the greatest number of cell lines and time points had the greatest number of upregulated genes in total. For example, geldanamycin increased expression of 35 genes, representing 38% of the 93 genes that were upregulated in the glomerulopathies, while neratinib increased only 9 genes or 10% (Supplementary Table S14). There were also considerable similarities in the upregulated genes among the drugs (Supplementary Table S14). Autophagy gene signatures were generally smaller compared with the ER/UPR genes (Supplementary Table S14). For example, neratinib increased expression of 11 genes, representing 10% of the 110 genes that were upregulated in the glomerulopathies (Supplementary Table S14).

We anticipated that the LINCS L1000 dataset would reveal ligands and kinases with gene signatures that would match the 93 ER/UPR or the 110 autophagy genes. Identification of various ligands or kinases could potentially have been helpful in validating the identified drugs. Surprisingly, there were only a few ligands (growth factors, cytokines) identified with signatures similar to the 93 ER/UPR genes (Supplementary Table S13), and the genes stimulated by these ligands included only HLA genes. Also, there was only a single protein kinase identified (NUAK1), with only four genes overlapping with the 93 ER/UPR genes (Supplementary Tables S13, S14).

The original CMAP drug database (“old CMAP”) was largely in keeping with LINCS L1000, as among candidate drugs, there were many Hsp90 inhibitors, as well as another EGFR inhibitor (Supplementary Table S13). This result strengthens the validity of the drugs selected *via* the L1000 dataset analysis. Importantly, thapsigargin, a drug that releases calcium from the ER and a well-known inducer of the UPR, was identified in the old CMAP drug database (Supplementary Table S13). The identification of thapsigargin, a “gold standard” ER/UPR drug, further supports the validity of the search method. Additional analysis of the thapsigargin gene datasets showed that the drug increased expression of 13 ER/UPR genes or 14% of the 93 genes that were upregulated in the glomerulopathies (Supplementary Table S14). Thus, the number of genes upregulated by the six L1000-ER/UPR/autophagy drugs is in the same range (or even greater) than the number upregulated by thapsigargin.

There were no ligands, kinases or additional drugs identified with signatures similar to the 110 autophagy genes (Supplementary Table S13). It should be noted that while neratinib was the single EGFR/HER2 inhibitor identified as matching both ER/UPR and autophagy gene signatures, four other EGFR inhibitor drugs showed significant matches with the autophagy signatures of the glomerulopathies (Supplementary Table S13), strengthening the validity of neratinib as a candidate autophagy-inducing drug.

Finally, since the above search involved genes in the JuCKD-Glom dataset, we also searched the LINCS L1000 database for candidate drugs using input of ER/UPR and autophagy genes upregulated in the other GN datasets (GSE108109, GSE115857 and GSE141295; Supplementary Table S1). As stated above, a number of ER/UPR and autophagy genes showed upregulation in GSE108109, GSE115857 and GSE141295, with modest-moderate overlap with JuCKD-Glom. There was considerable similarity between the L1000-ER/UPR/autophagy drugs selected based on JuCKD-Glom and the drugs selected using these other gene inputs. Specifically, geldanamycin, NVP-AUY922, radicicol, celastrol, withaferin-a and neratinib were also identified as candidate drugs based on GSE108109, GSE115857 and GSE141295 (Supplementary Table S13).

### L1000-ER/UPR/autophagy drugs stimulate expression of ER chaperones and/or proteins involved in autophagy

Four of the L1000-ER/UPR/autophagy candidate drugs were tested to see if they stimulate the UPR in cultured GECs. We examined the potential of the drugs to upregulate expression of the ER chaperones GRP94 (HSP90B1), BiP (HSPA5) and mesencephalic astrocyte-derived neurotrophic factor (MANF), which were previously shown to be upregulated as part of the UPR in GECs (12). After incubating GECs with geldanamycin, NVP-AUY922 and radicicol, we observed increases in GRP94, BiP and MANF proteins (most consistent with radicicol), although these increases, as expected, were not as robust as those induced by tunicamycin, a potent inducer of the UPR, used as a positive reference control (Figures 6A,C). As predicted, since these three drugs inhibit Hsp90, this leads to derepression of heat shock factor-1 and an increase in the cytosolic stress protein, Hsp70 (HSPA1A; Figures 6A,C). In an earlier study, we demonstrated that celastrol (another one of the L1000-ER/UPR/autophagy drugs) induces the UPR and Hsp70 in GECs (39). Neratinib did not increase GRP94, BiP or MANF protein expression; however, genes specifically encoding these three chaperones were not upregulated by neratinib, whereas neratinib upregulated another ER chaperone gene (ERP29), as well as XBP1, a transcription factor for multiple ER chaperones (Supplementary Table S14).

Expression of ER chaperones in the UPR is regulated transcriptionally, while autophagy is believed to be primarily a post-translational process; however, more recently it has become evident that transcriptional regulation of autophagy genes and their protein products may contribute to autophagy (7). Thus, we examined whether radicicol and neratinib can increase expression of proteins involved in autophagy, including LC3 (MAP1LC3B) and p62/SQSTM1 (12) (Supplementary Table S14). Incubation of GEC with neratinib (but not radicicol) increased total LC3 significantly, i.e., LC3-I + LC3-II, as well as p62 (Figures 6B,E), although, as with ER chaperones, the increases were not as robust as those induced by tunicamycin. To monitor autophagic flux, we incubated GECs with chloroquine, which blocks the fusion of autophagosomes with lysosomes and prevents autolysosomal protein degradation, allowing comparison of the rates of autophagosome formation. Neratinib+chloroquine increased LC3-II compared with chloroquine alone, in keeping with stimulation of autophagy (Figures 6B,E). LC3-II tended to be greater with radicicol, but the change did not reach statistical significance. Therefore, while neratinib did not stimulate expression of ER chaperones in GECs, the principal action of this drug was to stimulate autophagy proteins.

### Neratinib reduces GEC injury

First, we demonstrated that the L1000-ER/UPR/autophagy drugs were not toxic to GECs, using a LDH assay (Supplementary Table S12). In this assay, we included adriamycin, a drug known to injure podocytes and induce experimental FSGS in rodents *in vivo*, as well as podocyte ER stress and autophagy (12, 40). Low dose adriamycin (0.5 μM) did not induce LDH release.

In glomerulopathies, podocyte injury is generally not lethal or cytolytic (4). Cultured GECs form FACs, which mediate adhesion to extracellular matrix (2). To monitor injury, we used a GEC assay that quantifies FAC density based on vinculin expression. In this assay, dissolution of FACs correlates with injury, i.e., podocyte foot process effacement in primary FSGS *in vivo* (17, 32). We selected two drugs with putatively distinct primary targets (neratinib and radicicol) to test if the drugs could protect GECs from injury. We did not test celastrol in this assay, because in our previous study, celastrol proved to be toxic in mice *in vivo* (39). GECs were untreated, or were treated with low dose adriamycin (0.5 μM) to induce sublethal injury. In parallel, GECs were treated with neratinib or adriamycin+neratinib, as well as with radicicol or adriamycin+radicicol. Low dose adriamycin reduced the number of FACs (visualized by vinculin immunostaining), consistent with FAC dissolution due to sublethal injury (Figures 7A,B). Neratinib independently did not affect the number of FACs, and importantly, it attenuated the reduction in FACs induced by adriamycin by more than 50% (Figures 7A,B), implying that neratinib protected GECs from injury. Adriamycin and neratinib did not alter vinculin expression (Figure 7C), indicating that changes in the number of FACs were due to vinculin redistribution in cells. In the FAC assay, radicicol (0.5–1 μM) independently decreased FAC number, comparably to the effect of adriamycin shown in Figure 7B; thus, radicicol appeared to be toxic. A lower dose of radicicol (100 nM) did not independently reduce FAC number; however, this dose was not effective in attenuating FAC dissolution by adriamycin.

Finally, while neratinib was identified due to its induction of ER/UPR and autophagy gene signatures, it should be noted that the GECs used in this study express low levels of EGFR. This conclusion is based on mass spectrometry spectral counts, which are a semi-quantitative practical measure of protein abundance. The mean spectral counts of EGFR were 4.33 in untreated control GECs and 5.33 in KO GECs (3 measurements per group), reflecting low expression (13). HER2 spectral counts were not detected.

### IXA6, a drug directed at IRE1α, reduces GEC injury

In this set of experiments, we first verified that the UPR protects GEC from injury in the FAC assay. We compared injury in control (IRE1α-replete) GECs with IRE1α KO cells, in which activation of the UPR was shown to be impaired (12). Control and IRE1α KO GECs were untreated or were incubated with adriamycin (as above) to induce sublethal injury. By analogy to the results in Figure 7, the number of FACs (visualized by vinculin immunostaining) was reduced after adriamycin treatment; however, the reduction in FACs was almost 2-fold greater in IRE1α KO cells compared to IRE1α-replete control (Figures 8A,B). Therefore, it can be concluded that the IRE1α UPR pathway is protective.

Next, we used control (IRE1α-replete) GECs to examine if IXA6, a drug shown to stimulate the IRE1α RNase (15), could attenuate GEC injury. GECs were untreated, or treated with adriamycin to induce sublethal injury. In parallel, GECs were treated with IXA6 or adriamycin+IXA6. IXA6 independently did not affect the number of FACs, and the drug significantly attenuated the reduction in FACs induced by adriamycin (Figures 8C,D), indicating that IXA6 was cytoprotective. Finally, although the action and specificity of IXA6 toward IRE1α was already characterized extensively (15), we verified that IXA6 activates the IRE1α pathway in GECs. Incubation of control GECs with IXA6 increased expression of MANF, an ER chaperone that we previously showed is activated *via* the IRE1α pathway in GECs (12). IXA6 did not affect expression of GRP94, a chaperone that is more dependent on the activation of the ATF6 pathway, nor as expected, expression of Hsp70 (Figures 8E,F).

## Discussion

Analysis of gene expression in human GN (MN, FSGS and IgAN) and DN demonstrated that there are many common upregulated glomerular genes, including genes related to the ER/UPR and autophagy. Postulating that activation of the UPR and autophagy are mechanisms that are cytoprotective in glomerular diseases, we employed connectivity mapping to identify drugs that can potentially activate these pathways. These drugs included inhibitors of Hsp90 or EGFR/HER2. Candidate drugs were then shown experimentally to induce the UPR or autophagy in cultured GECs. Finally, in a glomerular cell culture assay that correlates with glomerular damage *in vivo*, one candidate drug – neratinib (EGFR/HER2 inhibitor) was shown to be cytoprotective.

Our analysis of the JuCKD-Glom dataset demonstrated that ~2000 genes were upregulated in glomeruli, and interestingly, more than 1,000 of these genes were common to MN, FSGS and IgAN. GO analysis revealed a number of pathways associated with ER components, ER function and the UPR, and a smaller number of pathways associated with autophagy. The majority of these pathways were present in all three GNs. We then developed sets of genes associated with the ER/UPR and autophagy, and showed that ~90% of genes in ER/UPR and autophagy protein–protein interaction networks were present in our gene sets. Analysis of the ER/UPR and autophagy genes in MN, FSGS and IgAN showed that these genes were upregulated in all of these GNs, and 35–45% of these genes were common to the three GNs.

One limitation of our analyses is that the JuCKD-Glom dataset contains a relatively small number of patients with GNs. However, analyses of glomerular gene expression in three additional GN datasets confirmed activation of ER/UPR and autophagy pathways. In these additional datasets, a substantial number of ER/UPR and autophagy genes showed increased expression, and while the overlap of specific genes with those observed in JuCKD-Glom was only modest-moderate, a substantial majority of the ER/UPR and autophagy-related GO biological pathways and gene categories overlapped with JuCKD-Glom. DN is likely more heterogeneous in pathogenesis compared with GN, and only a limited number of patients with DN undergo kidney biopsies. Nevertheless, analysis of a small number of DN biopsies in JuCKD-Glom showed increased expression of ER/UPR and autophagy genes, and almost all of these genes were also increased in the three GNs.

MN, FSGS and IgAN have distinct pathogenic mechanisms and there is variability in the primary cellular targets of injury in the glomerulus. Despite this, the three GNs share many upregulated genes, and specifically, all show increases in genes associated with activation of ER stress and the UPR, as well as autophagy. Furthermore, single cell RNA sequencing analysis of glomerular cells shows that basal expression of the upregulated ER/UPR and autophagy genes is not restricted to a single glomerular cell type. These results suggest that despite distinct pathogenic mechanisms believed to initiate these GNs, there may be significant commonalities in pathways that are activated to mediate glomerular injury, and common pathways that attenuate injury include the UPR and autophagy.

When considering therapeutic approaches to GN, a key question is whether one searches for drugs that might be therapeutic in all of these diseases, or is each disease an entirely separate entity? Our gene expression analysis suggested that common pathways, i.e., UPR and autophagy, may potentially be targeted with drugs. We approached this using connectivity mapping, which aims to identify candidate drugs for a disease by relating the gene expression signatures of different drugs with that of the disease. Thus, given the GN-associated ER/UPR and autophagy gene signatures, and the premise that activation of the UPR and autophagy are mechanisms that are cytoprotective in glomerular diseases (7), we used the LINCS L1000 dataset to interrogate which drugs induce gene expression signatures in cultured cell lines that are similar to those in GN. The rationale is that candidate drugs stimulating the UPR or autophagy when given to patients with GN would further stimulate the endogenous UPR or autophagy, or provide for more sustained activation of these processes, and thereby enhance cytoprotective mechanisms, reduce protein misfolding and improve proteostasis (15). At the same time, such drugs should not trigger overwhelming ER stress that would lead to toxicity. Our search resulted in the identification of multiple drugs that induced ER/UPR or autophagy gene signatures in cell lines. Among these, six drugs appeared to stimulate both ER/UPR and autophagy gene signatures, and for which descriptions were available in the literature (see below). We considered the latter to be an important criterion for selection of candidate drugs for further study, since description in the literature implies that the drug is either already FDA-approved or has been tested *in vivo*, thereby facilitating potential “repurposing” for eventual treatment of GN. The six candidate drugs were identified using glomerular gene expression signatures from several distinct datasets, and they included Hsp90 inhibitors, a EGFR/HER2 inhibitor and drugs with multiple potential actions. We attempted to substantiate the validity of our drug search by examining for potential growth factors, cytokines or protein kinases that would induce signatures similar to GNs and drugs in cultured cells; however, there were very few candidates, and this approach was not useful. A limitation of the LINCS L1000 dataset is that although it contains immortalized kidney cell lines, there are no primary glomerular cell lines in this dataset, and the large majority of cell lines are immortalized tumor cells. In addition, the L1000 technology measures expression for 978 “landmark” genes directly, while the expression values of the remaining transcriptome is estimated using a computational model (23).

We proceeded to show experimentally that three drugs, which are categorized as Hsp90 inhibitors (geldanamycin, radicicol and NVP-AUY922) increased protein expression of three ER chaperones in cultured GECs, in keeping with the activation of the UPR. Earlier, we showed that celastrol was also able to activate the UPR *in vivo* (39). The Hsp90 inhibitors did not, however, stimulate significant increases in proteins involved in autophagy. In contrast, neratinib did not increase expression of the three ER chaperones we analyzed, but neratinib increased expression of LC3 and p62 proteins, in keeping with the ability of this drug to upregulate autophagy genes. Thus, the drugs identified as stimulators of the UPR or autophagy genes using connectivity mapping were, at least in part, validated experimentally.

The ultimate goal after discovering cytoprotective drugs by connectivity mapping is to demonstrate their protective effect on cell injury experimentally. For this, we used a validated GEC culture assay that quantifies the density of FACs based on vinculin expression (17, 32). We induced injury of GECs with a low dose adriamycin, which was reflected as dissolution of FACs, (a surrogate measure of podocyte foot process effacement *in vivo*). When GECs were treated in parallel with neratinib, the adriamycin-induced dissolution of FACs was attenuated significantly. The Hsp90 inhibitor radicicol, however, proved to be independently toxic in this assay. Thus, drugs that inhibit EGFR, such as neratinib, deserve further consideration for therapy of GN. Using the FAC assay, we also demonstrated that the IRE1α UPR pathway is critically important in protecting cells from adriamycin-induced injury, as deletion of IRE1α exacerbated adriamycin-induced dissolution of FACs. Furthermore, stimulation of the IRE1α RNase with IXA6 attenuated the adriamycin-induced reduction in FACs, supporting the view that stimulation of the UPR/autophagy is protective and a viable option for potential therapy for GN. In the future, our results in cell culture models will require corroboration *in vivo*.

Some of the drugs that we identified in this study by connectivity mapping or drugs with analogous mechanisms of action have been examined in a few animal models of glomerular disease. For example, a Hsp90 inhibitor ameliorated high fat diet-induced renal failure in diabetes (41) and lessened disease in the MRL/lpr mouse model of systemic lupus erythematosus (42). The EGFR inhibitor erlotinib was associated with a reduction in albuminuria and glomerular injury in diabetic nephropathy and an increase in autophagy in the kidney (43, 44). An EGFR inhibitor also ameliorated experimental mesangial-proliferative GN (45). Celastrol improved insulin resistance and attenuated renal injury in db/db diabetic mice (46); however, in our experience, celastrol proved to be toxic and ineffective in a mouse model of FSGS (39). Given that we identified neratinib through transcriptional signatures similar to the ER/UPR or autophagy, an intriguing possibility that arises from these results is that the cytoprotective effects of neratinib or other EGFR inhibitor drugs can be attributed to activation of the UPR and/or autophagy rather than blocking EGFR signaling. Actually, autophagy and EGFR signaling may be linked, as EGFR activation was reported to downregulate autophagy *via* phosphorylation of Beclin-1 (47). Future studies will be required to address the specific mechanisms involving the cytoprotection of EGFR signaling inhibitors.

Before effectively administering drugs *in vivo*, there is a need to establish non-invasive biomarkers to diagnose ER stress and to monitor drug therapy. Monitoring excretion of certain ER chaperones into the urine may be one approach (48, 49). Such approaches will allow the ongoing monitoring of mechanistic parameters of drug therapy in addition to drug effectiveness. Our “proof of concept” study is timely, since excellent drug targets for various aspects of podocyte function are emerging, and a number of these drugs are already FDA-approved (7, 50). By providing additional evidence that ER stress/UPR and autophagy are components of GN, the present study opens the possibility of modulating ER stress/autophagy pharmacologically as therapy for GN.

## Data availability statement

The datasets presented in this study can be found in online repositories. The names of the repository/repositories and accession number(s) can be found in the article/Supplementary material.

## Author contributions

AC, C-FC, and AE conceived and designed the research. C-FC, JN-B, AB, AE, and AC analyzed the data. JP and JG performed experiments. AC wrote the manuscript. All authors contributed to the article and approved the submitted version.

## Funding

This work was supported by Research Grants from the McGill Initiative in Computational Medicine, Canadian Institutes of Health Research (MOP-133492, PJ9-166216, and PJ9-169678), the Kidney Foundation of Canada, and the Catherine McLaughlin Hakim Chair.

## Conflict of interest

The authors declare that the research was conducted in the absence of any commercial or financial relationships that could be construed as a potential conflict of interest.

## Publisher’s note

All claims expressed in this article are solely those of the authors and do not necessarily represent those of their affiliated organizations, or those of the publisher, the editors and the reviewers. Any product that may be evaluated in this article, or claim that may be made by its manufacturer, is not guaranteed or endorsed by the publisher.

## Supplementary material

The Supplementary material for this article can be found online at: https://www.frontiersin.org/articles/10.3389/fmed.2023.1122328/full#supplementary-material

## Footnotes

^1^ https://lincsproject.org/LINCS/tools

^2^ https://www.networkanalyst.ca
