# Supplementary figures and images for: Analysis of gene expression and use of connectivity mapping to identify drugs for treatment of human glomerulopathies
Source: Front Med (Lausanne). 2023 Mar 13;10:1122328. doi: 10.3389/fmed.2023.1122328 (PMC10042326; doi:10.3389/fmed.2023.1122328)

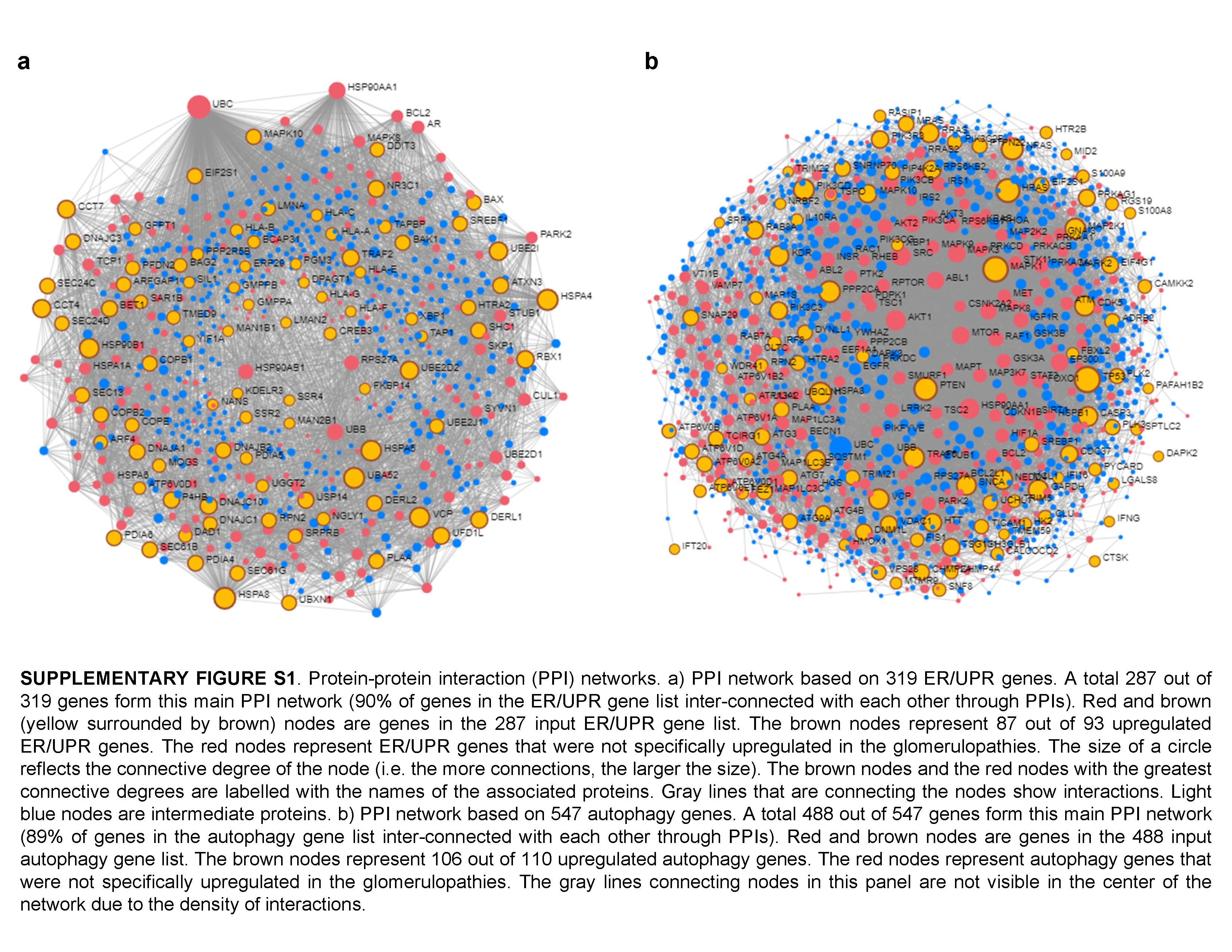

Supplement: Supplementary file 15 [file Image_1.tif]
